# CD8+ cell somatic mutations in multiple sclerosis patients and controls—Enrichment of mutations in STAT3 and other genes implicated in hematological malignancies

**DOI:** 10.1371/journal.pone.0261002

**Published:** 2021-12-07

**Authors:** Miko Valori, Lilja Jansson, Pentti J. Tienari

**Affiliations:** 1 Translational Immunology Research Program, University of Helsinki, Helsinki, Finland; 2 Department of Neurology, Neurocenter, Helsinki University Hospital, Helsinki, Finland; CNR, ITALY

## Abstract

Somatic mutations have a central role in cancer but their role in other diseases such as common autoimmune disorders is not clear. Previously we and others have demonstrated that especially CD8+ T cells in blood can harbor persistent somatic mutations in some patients with multiple sclerosis (MS) and rheumatoid arthritis. Here we concentrated on CD8+ cells in more detail and tested (i) how commonly somatic mutations are detectable, (ii) does the overall mutation load differ between MS patients and controls, and (iii) do the somatic mutations accumulate non-randomly in certain genes? We separated peripheral blood CD8+ cells from newly diagnosed relapsing MS patients (n = 21) as well as matched controls (n = 21) and performed next-generation sequencing of the CD8+ cells’ DNA, limiting our search to a custom panel of 2524 immunity and cancer related genes, which enabled us to obtain a median sequencing depth of over 2000x. We discovered nonsynonymous somatic mutations in all MS patients’ and controls’ CD8+ cell DNA samples, with no significant difference in number between the groups (p = 0.60), at a median allelic fraction of 0.5% (range 0.2–8.6%). The mutations showed statistically significant clustering especially to the *STAT3* gene, and also enrichment to the *SMARCA2*, *DNMT3A*, *SOCS1* and *PPP3CA* genes. Known activating *STAT3* mutations were found both in MS patients and controls and overall 1/5 of the mutations were previously described cancer mutations. The detected clustering suggests a selection advantage of the mutated CD8+ clones and calls for further research on possible phenotypic effects.

## Introduction

Somatic mutations can occur early or late in life and lead to mosaicism in different cell lineages. The mutation rates differ between tissues, ranging from 3.5 x 10^−9^ (small intestine) to 1.6 x10^-7^ (skin) mutations per base-pair [1]. Blood cells accumulate mutations during lifetime and mutations that provide a survival advantage or promote proliferation will enrich by time in a process called clonal hematopoiesis. Somatic mutations have been shown to increase in number during aging. In population cohorts not selected for cancer or hematologic phenotypes somatic mutations in whole blood DNA with allelic fraction of 10–20% have been found in approximately 1% of subjects under age 50, and in 10% of subjects older than 70 years [2, 3].

Somatic mutations have an established role in cancer; their role in non-malignant diseases is also gaining attention. Single cell sequence analyses have raised the hypothesis that somatic mutations may modulate brain aging and neurodegeneration [4]. Activating *KRAS* mutations have been observed in arteriovenous malformations in the brain [5]. There are also examples of rare autoimmune diseases in which somatic mutations play a role. In children with an autoimmune lymphoproliferative syndrome, somatic mutations in *FAS* have been discovered, and these mutations induced a defect in T-cell apoptosis [6]. Similar childhood autoimmune disease has also been described with activating somatic mutations in the *KRAS* gene, which also impair T-cell apoptosis [7]. Recently, somatic mutations in X-chromosomal *UPA1* gene were reported in men with an adult-onset inflammatory syndrome called VEXAS (vacuoles, E1 enzyme, X-linked, autoinflammatory, somatic) [8].

The role of somatic mutations in more common autoimmune disorders is not clear. Activating *JAK1* and *STAT3* mutations have been discovered in the gut intraepithelial lymphocytes that underwent malignant transformation in refractory celiac disease [9]. Another example in the border-zone of cancer and autoimmunity is the discovery of activating *STAT3* mutations in CD8+ cells of patients with large granular lymphocyte (LGL) leukemia and rheumatoid arthritis [10, 11]. In a subsequent analysis of 25 newly-diagnosed rheumatoid arthritis patients and 20 controls *STAT3* mutations were not found, but other somatic mutations were discovered in the expanded CD8+ effector-memory subset in 20% of patients vs. 5% of controls [12].

Multiple sclerosis (MS) is a chronic inflammatory disease of the central nervous system and among the most common causes of neurological disability in young adults. In relapsing MS, which constitute about 90% of cases, there is evidence based on genetics, environmental risk factors and treatment paradigms that peripheral leukocyte dysfunction plays a major role in the disease [13–15]. Inflammation in MS occurs in plaques, and analyses of rare cases of acute plaques have identified clonal expansion of CD8+ T cells [16]. Recent analyses of MS patient’s T-cell receptor Vβ repertoire suggest that the CD8+ clones present in MS plaques can be detected in the cerebrospinal fluid and blood [17].

Indirect evidence of somatic mutations in MS patients’ cultured T-lymphocytes was reported in 1990s by using the hypoxanthine guanine phosphoribosyltransferase (HPRT) assay. This assay measures 6-thioguanine resistance of cultured cells, caused by inactivating somatic mutations of the X-chromosomal HPRT gene or other mechanisms. MS patients’ HPRT-deficient T-lymphocyte clones, but not wild-type clones, were potentially autoreactive, i.e. proliferated in response to myelin autoantigen [18] and a higher HPRT-deficient T lymphocyte frequency was reported in MS patients as compared to controls [19]. A pilot study on amplified DNA of CD4+ cells, derived from two MS patients’ CSF reported thousands of mutations in an exome-wide analysis of 21 individual cells. These mutations were considered PCR amplification artifacts and this study points to important technical limitations, when small amounts of DNA are amplified [20]. We and others have subsequently demonstrated that nonsynonymous somatic mutations are detectable in MS patients blood cells in about 60% of cases and that the mutant clones persist over time [21–23]. Mutations were predominantly (85%) found in the CD8+ fraction [21].

Here we extend these findings by using an improved methodology for mutation discovery and analyze a cohort of 21 newly-diagnosed MS patients and 21 matched controls. The comparison between groups allows us to form an initial picture of whether the previously reported findings in MS are disease specific or not. Another major goal enabled by the sample size (n = 42 participants total) is an assessment of non-random accumulation of somatic mutations in certain genes, which we carry out in addition to the case-control comparison. Because the detection of somatic variants in small cell clones is very dependent on sequencing depth, we opted to limit our search to a subset of the exome containing key immunological and cancer-related genes, which allowed us to reach high sequencing depths of over 2000x and obtain a more detailed view over small allelic fraction events.

## Materials and methods

### Study participants

Patients (n = 21) were recruited at the Helsinki University Hospital Department of Neurology outpatient clinic during their diagnostic examinations and were selected from a larger collection of patients to fulfill the inclusion criteria and age- and sex-matching. Inclusion criteria for MS patients were: 1. McDonald 2010 criteria for relapsing MS, 2. CSF oligoclonal bands and 3. Barkhof’s magnetic resonance imaging criteria (20 patients fulfilled 4/4 criteria one patient fulfilled ¾) for anatomical dissemination of demyelination [24]. The location of presenting symptom was spinal in 9, brainstem in 4, optic neuritis/hemispheral in 5 and multifocal in 3 patients. Their subsequent treatments were the following: injectables in 5 (beta-interferons and glatiramer acetate), oral in 13 (teriflunomide, dimethyl fumarate, fingolimod) and infusions in 2 (natalizumab, rituximab). One patient did not want to start any treatment. None of the patients had started disease-modifying treatment when the blood sample was taken. Age- and sex-matched controls (n = 21), free of autoimmune disease or cancer, were selected from other neurological diseases visiting the outpatient clinic and from staff of the campus. The demographic features of the patients and controls are shown in Table 1 and more detailed data in S1 Table (MS patients) and S2 Table (controls). This study has been approved by the regional ethics committee (Dno 83/13/03/01/2013). All participants gave informed written consent.

**Table 1:** Demographic features of the participants.

	Relapsing MS patients	Controls
**Number**	21	21
**Mean age (range)**	35.0 yrs (23–55)	35.2 yrs (23–57)
**Percentage females**	76%	76%
Baseline EDSS* **Mean (range)**	1.64 (0–4.5)	n.a.

*Expanded disability scale score (EDSS) at first neurologist visit.

### CD8+ cell separation and DNA extraction

Peripheral blood mononuclear cells (PBMCs) were extracted from 120–140 ml venous EDTA blood using Ficoll-Paque PLUS (GE Healthcare). First, 13 ml of Ficoll-Paque was added to a centrifuge tube. Then, 9 ml of blood diluted with 28 ml of PBS was layered on top of it. The tube was centrifuged at 800 x g for 30 minutes after which the PBMC layer was transferred to a new tube with a pipette. The cells were washed twice, using PBS and centrifugation at 500 x g for 15 minutes and at 500 x g for 10 minutes. From the PBMCs, positive separation with MACS CD8 antibody MicroBeads (catalogue number130-045-201, Miltenyi Biotec, Bergisch Gladbach, Germany) was performed using an OctoMACS magnetic separator (Miltenyi Biotec) following the manufacturers protocol. We simultaneously separated also other cells, which will be analyzed later. From the separated cell populations, DNA and RNA were extracted using the InviTrap Spin Universal RNA Mini Kit (Stratec Biomedical, Birkenfeld, Germany) according to manufacturer’s instructions. The purities of the separated CD8+ cells were tested in 31 (74%) of the 42 of samples by flow cytometric analysis, in which T cells (CD3+) represented 89–99% of the cells and the observed purities for CD8 were all ≥ 87%. T-cell receptor Vβ repertoire was not included to the analyses, because in our previous analysis the presence of a large Vβ clone did not significantly predict the detection of a somatic mutations with small allelic fractions [21].

### Target genes and sequencing

A gene panel that consists of 2524 genes related to immunity and cancer (Immunopanel-2524) was designed for mutation screening [25]. The gene list is given in S3 Table. DNA (1000 ng) from the separated cell populations was fragmented using a Covaris S2 instrument (Covaris,Woburn, MA, USA) and then a sequencing library was prepared according to the NEBNext DNA Sample Prep Master Mix Set 1 (New England BioLabs, Ipswich, MA, USA). Target enrichment for all coding exons (target size ca. 5 Mb) was carried out with the Nimblegen SeqCap exon capture system (Roche NimbleGen). Resulting library was sequenced with a HiSeq 2500 instrument (Illumina) using 150bp paired end reads. A high sequencing depth (overall median 2349x, range 1460x-3534x, MS cases median 2317x & range 1524x-3534x, controls median 2381x & range 1460x-3220x) was attained for the sequenced samples (S1 Fig), to facilitate the detection of very low allelic fraction events.

A “reference DNA” pool was collected from 8 whole blood DNAs of healthy donors, to act as technical control material. These DNA samples were sequenced in an identical manner to a combined median depth of 4343x. The samples for the reference DNA pool were obtained via Meilahti Biobank from individuals aged 20–35 years without hematological or autoimmune conditions.

### Data analysis

The sequencing reads were trimmed for adapters and base quality using Trimmomatic [26], after which they were mapped to the GRCh37 reference genome with BWA MEM [27]. PCR duplicates were removed using Samblaster [28]. Somatic variant calling was performed with Tnscope [29], using a pool of 8 whole blood samples as a technical reference to discard common sequencing, library preparation and mapping artefacts. The separated CD8+ cell fraction of a participant was used as the “tumor” bam file for TNScope, and the whole blood pool reference as the “normal” bam file. TNscope was set to only consider bases of quality score 30 or higher. Variant annotation was performed using ANNOVAR [30].

The somatic variant calls were filtered using several methods. TNScope produced VCF filter statistics were checked, and suspect artefact or possible germline calls were removed from each sample’s data. Germline variant calls were removed within-sample based on a call’s allelic fraction, and germline variants detected in any of the other samples were also removed to avoid possible cross-contamination. Furthermore, somatic variants were only called at high depth (>100x) locations in order to facilitate accurate calculation of the allelic fraction and to avoid germline mix-up. Strand bias filtering was used to discard systematic sequencing errors that preferably show up in one sequencing direction only. Known segmental duplication areas were excluded from the analysis to avoid errors from mismapping of pseudogenes at these loci. Errors from mismapping of reads were mitigated by discarding highly clustered variants and by requiring a similarly high mapping quality from variant reads as from the reference allele containing reads. Variants with a frequency of >10^−5^ in the ExAC or gnomAD germline population databases were also filtered to remove remaining possible germline contamination and to exclude common mapping errors and other artefacts. To remove common erroneous variant calls, the ExAC and gnomAD database variant lists that were used were purposefully of the unfiltered type i.e., they included even the population variant calls that don’t pass the quality control filters of ExAC or gnomAD, and likely represent repeating artefacts.

In order to automatically calibrate the variant calling sensitivity level against several classes of library preparation related base substitution errors, an empirical trinucleotide-context aware noise frequency was calculated for each possible base substitution, separately for each sample. The noise frequencies were calculated from the data by counting the number of base mismatches against the reference genome, for all sequenced coordinates, excluding germline variant sites, tabulated separately for each possible 192 trinucleotide context and base substitution combinations. Using the empirical noise frequencies calculated this way, a binomial test that takes into account the number of variant observations, the sequencing depth, and the noise frequency was used to discard likely library preparation related artefacts, by requiring more evidence at noisier locations.

We paid especial attention to base changes of type C>T (G>A) because previous studies have indicated a risk of erroneous C>T (G>A) substitutions arising during library preparation [31]. These are caused by deamination of cytosine residues, typically at CpG dinucleotides, which are often in the methylated state [32]. Artifact substitutions of this class can even be generated by thermal cycling without any DNA synthesis [31]. In our data-derived trinucleotide context aware noise estimates, abundant C>T (G>A) reference genome mismatches were present an order of magnitude more often than most other substitutions, especially when encountered in a CpG sequence context (S4 Table). To limit the effect of C>T (G>A) and other false positives in our results, we chose to set our sensitivity cutoff favoring specificity so that the base distribution of the somatic variant calls remained similar to the germline variant calling performed on the same data. Increasing sensitivity beyond this point to pick up events at even smaller allelic fractions was found to greatly increase the rate of C>T (G>A) somatic variant calls in comparison to the baseline germline rate. These we considered likely false positives because we assume that the somatic mutations present at higher variant allele fractions should follow a similar base distribution to those at modestly lower allelic fractions rates, instead of an increasing C>T (G>A) bias for the lower allelic fraction calls that are supported by less evidence (number of sequencing reads).

After filtering for quality, all synonymous variants were excluded (n = 160) from detailed analysis. The variant list was also filtered by excluding genes that are not expressed in CD8+ PBMCs according to RNA-Seq data that we had previously generated [21], which resulted in removal of 76 mutations in MS patients and 88 in controls. See S5 Table for the full list of variant filtering procedures applied. Deleteriousness of the somatic variants was assessed using the CADD [33] and PolyPhen [34] algorithms, with a CADD score of > 20 considered possibly deleterious. Enrichment of somatic mutations on certain genes was assessed using a Poisson test, where the per-gene expected mutation rate parameter was obtained from the size of each gene, the size of the full gene panel target area, and the total number of mutations found in all participants. Synonymous somatic variants were evaluated using the TraP [35] and SURF [36] algorithms.

### Amplicon sequencing

As a quality control measure, targeted PCR amplicons from CD8+ cell DNA were designed to verify some of the discovered variants. The amplicons were sequenced using Illumina MiSeq with 2x300bp reads. Variant loci were inspected using samtools mpileup and base counts were compared to a control DNA amplified in the same manner.

## Results

### Somatic mutations with low allelic fraction are discovered in all donors in CD8+ T cells

We were able to perform unusually high depth sequencing of over 2000x median coverage for 42 CD8+ cell DNA samples. All of the sequenced CD8+ samples were found to contain high-confidence somatic variants. After quality filtering, and including synonymous variants, the number of detected somatic mutations was 652 (median and mean numbers of mutations per subject were 13.5 and 15.5). The total number of mutations was 307 in MS patients (median 13, mean 14.6, range 3–41 per subject) and 345 in controls (median 14, mean 16.4, range 4–35 per subject). After removing synonymous variants and variants in genes not expressed in CD8+ cells a total of 225 nonsynonymous somatic mutations were detected in genes expressed in CD8+ cells.

**Fig 1:**
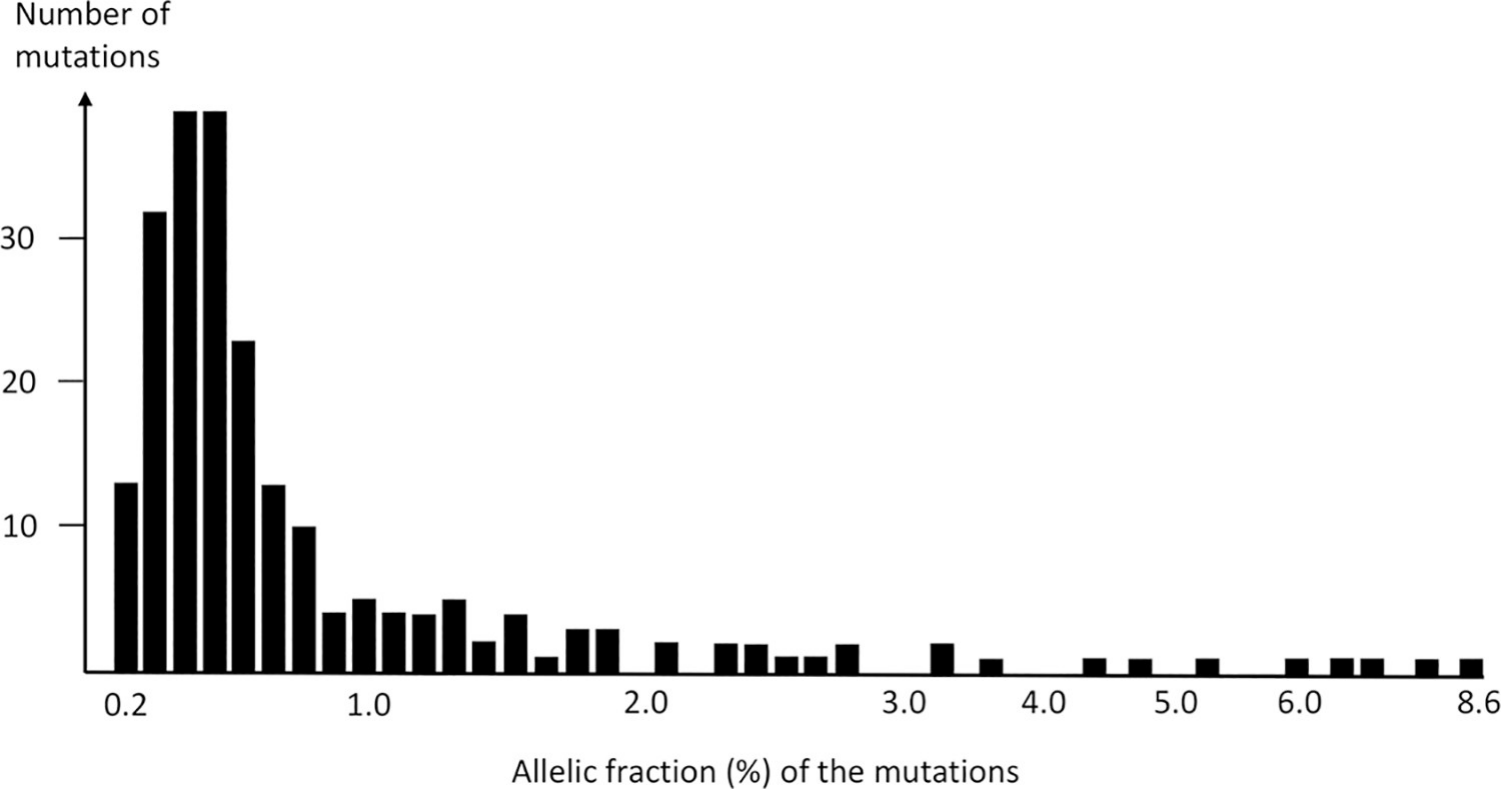
Participants’ CD8+ cell somatic mutation allelic fraction distribution.

The number of nonsynonymous mutations in genes expressed in CD8+ cells was 104 in MS patients and 121 in controls (Mann-Whitney U-test, p = 0.60). A comparison of mutations between cases and controls is shown in Table 2. The majority of the mutations were predicted to affect the function of the gene product in both groups and all participants had at least one such mutation. Mutation type was classified as (i) predictably deleterious single nucleotide variation (both CADD and PolyPhen2 algorithms), (ii) stop-codon, (iii) frameshift insertion/deletion, or (iv) splice-site mutation in 62 (60%) of the mutations found in cases and in 69 (57%) of the mutations in controls (Table 2). The distribution of the mutations by allelic fraction is illustrated in Fig 1. As in our previous study (20), most of the mutations (77%) were found in allelic fractions of <1%.

**Table 2:** Comparison of the discovered nonsynonymous somatic mutations in genes expressed in CD8+ cells in MS patients and controls.

	MS patients (n = 21)	Controls (n = 21)
Median sequencing depth	2317	2381
Total number of mutations	104	121
Number of mutations per subject (median)	4	4
Number of mutations per subject (range)	1–11	1–15
Mutation allelic fraction (median)	0.5%	0.5%
Mutation allelic fraction (mean)	0.7%	1.1%
Mutation allelic fraction (range)	0.2–3.6%	0.2–8.6%
Number of mutated genes	95	107
Novel mutation	63 (61%)	82 (68%)
Mutation found in COSMIC database	23 (22%)	23 (19%)
Mutation found in ExAC/gnomAD (AF<10–5)	18 (17%)	16 (13%)
**Mutation type:**		
Stop codon	9 (8.7%)	8 (6.6%)
Frameshift insertion/deletion	4 (3.8%)	13 (10.7%)
Non-frameshift insertion/deletion	1 (1.0%)	7 (5.8%)
Splice site mutation	0	2 (1.7%)
Single nucleotide variation	90 (86.5%)	91 (75.2%)
CADD ≥20	60/89* (67%)	67/90* (74%)
PolyPhen2 D+P	49/89 (55%)	46/90 (51%)
PolyPhen Benign	40/89 (45%)	44/90 (49%)

COSMIC = Catalogue of Somatic Mutations in Cancer. AF = allele frequency, CADD = Combined Annotation Dependent Depletion, CADD scores ≥20 can be considered predictably deleterious. PolyPhen2 D = deleterious, P = probably deleterious. *One nonsynonymous SNV could not be classified by CADD and PolyPhen21.

The 225 nonsynonymous somatic mutations in genes expressed in CD8+ cells were found in 186 different genes and the mutations mostly did not recur between samples. Three mutations were found in two participants (IGFR2*G712Dl, TAB3*P182del, TRPV2*T96G), in one MS patient and in one control. Otherwise, the mutations were singleton observations.

### Age and gender effects

Since aging is associated with the number of somatic mutations in most tissues, we tested whether there would be an association between age and the number of mutations (all mutations included). There was an association between participant age and the number of somatic mutations, when normalized by the sequencing depth (p = 0.01, S2 Fig). Age also associated with the allelic fraction of the mutations (surrogate of clone size), the mean allelic fractions in subjects 23–29 yrs vs. 41–57 yrs were 0.71% vs. 1.24% (Mann-Whitney U-test, p<0.001).

The number of detected somatic mutations was spread similarly between males and females, with no statistical difference detected between these groups (Mann-Whitney U-test p = 0.36). As expected, the median sequencing depth had a modest association to the number of somatic mutations detected in that sample (Kendall’s tau p = 0.02).

### Most commonly mutated genes: STAT3, SMARCA2, SOCS1, DNMT3A, PPP3CA

We analyzed enrichment of nonsynonymous mutations to specific genes. Because a large coding region size of a gene increases the likelihood of mutations, we calculated Poisson p-values that take into account the size of each gene, the number of mutations in that gene, the total gene panel area, and the total number of mutations. There were 17 enriched genes that were mutated in two or more participants at p<0.05 (Table 3).

**Table 3:** Genes enriched with somatic mutations.

Gene	Enrichment p-value	Participants with mutation	Cases with mutation	Controls with mutation
STAT3	9.00E-08	6	2	4
SMARCA2	0.001060894	4	2	2
SOCS1	0.001550647	2	1	1
DNMT3A	0.002299316	3	3	0
PPP3CA	0.008899261	2	2	0
CASP10	0.011354062	2	2	0
IQCB1	0.011560434	2	1	1
ARAF	0.012015277	2	1	1
IGSF8	0.012114642	2	0	2
RFX5	0.012226858	2	1	1
PIAS3	0.01268028	2	1	1
TAB3	0.016054429	2	1	1
TRPV2	0.018313686	2	1	1
TNFAIP3	0.019490645	2	1	1
VLDLR	0.023452248	2	2	0
PDE3B	0.036479269	2	0	2
NCKAP1L	0.037371546	2	2	0

The enrichment p-value is obtained using a Poisson test, where it is calculated using the length of each gene and the number of mutations (out of total 225 possible) found in that gene, and the total size of the gene panel.

Interestingly, the *STAT3* gene stood out as the most enriched locus (p = 9 x 10^−8^) with a total of 6 nonsynonymous somatic mutations (2 in cases and 4 in controls, Table 3). *STAT3* is a gene known to be mutated in various hematological malignancies, and some of the mutations that we found were previously known activating mutations (D661Y and S614R each in one MS patient, Y640F and H410R each in one control, Table 4).

**Table 4:** Top 5 mutated genes.

Subject	Age	Gender	Mutation	CADD score	Found in COSMIC	Allelic fraction
MS-4	48 yrs	F	STAT3*D661Y	34	Yes	0.5%
MS-8	52 yrs	M	STAT3*S614R	25.5	Yes	0.4%
Control-55	39 yrs	F	STAT3*Y640F	24.2	Yes	1.9%
Control-31	30 yrs	F	STAT3*H410R	26.6	Yes	1.1%
Control-3	57 yrs	F	STAT3*A596V	23.6	-	0.4%
Control-45	28 yrs	M	STAT3*R278L	35	-	0.5%
MS-20	26 yrs	F	SMARCA*G366D	25.7	-	0.4%
MS-44	34 yrs	F	SMARCA*P153S	23.7	-	0.6%
Control-7	54 yrs	M	SMARCA*D208Y	24.4	-	0.8%
Control-25	28 yrs	F	SMARCA* Q228Rfs	n.a.	-	1.3%
MS-24	32 yrs	F	SOCS1*F148L	23.2	Yes	3.4%
Control-21	47 yrs	F	SOCS1*P97_G99del	n.a.	-	4.6%
MS-2	34 yrs	F	DNMT3A* M700Hfs	n.a.	-	0.5%
MS-14	23 yrs	M	DNMT3A*R214S	32	Yes	0.8%
MS-50	55 yrs	M	DNMT3A*G494W	35	-	1.2%
MS-46	30 yrs	M	PPP3CA*R426Q	22.9	Yes	0.3%
MS-52	41 yrs	F	PPP3CA* T458R	24.1	-	0.7%

F = female, M = male. CADD scores ≥20 can be considered predictably deleterious. COSMIC = Catalogue of Somatic Mutations in Cancer. fs = frameshift. Note: Only one SMARCA2 variant listed in control-25 and one SOCS1 variant listed in MS-24. Two insertion-deletion variants were found at the SMRCA2 site (rs757850599 and rs753013339), but these are not necessarily independent variants due to their occurrence in a CAG-repeat. Their frequencies in ExAC are 0.000032 (rs757850599) and 0.000031 (rs753013339) and allelic fractions were 1.2% and 1.3% in our data. The SOCS1*F148L variant occurred together with a frameshift SOCS1*E149Rfs*57 variant.

The top 5 mutated genes (p<0.01) and their mutations in cases and controls are shown in Table 4 in more detail. In addition to *STAT3*, these included *SMARCA2*, a tumor suppressor involved in various cancers, *SOCS1*, a STAT-inhibitor, *DNMT3A*, with known somatic mutations in the context of clonal hematopoiesis and *PPP3CA* encoding calcineurin-A, involved in T-cell receptor signaling.

All 225 nonsynonymous somatic mutations in genes expressed in CD8+ cells, their allelic fractions, base counts, presence in databases, discovery p-values and predicted deleteriousness are shown in S6 Table.

### Testing variant filtering with a variant of type C>T (G>A)

To test our somatic variant calling sensitivity and specificity settings (see 2.4 Data analysis), we prepared confirmation PCR amplicons for the two high-confidence *STAT3* somatic mutations detected in our MS patients (D661Y and S614R variants). For comparison we chose a low-confidence but recurring C>T variant ITPR3*T2584M, which was filtered out in our pipeline. A more relaxed sensitivity level would have called this variant in three MS patients and one control. This variant call represented a potential mutational hotspot, and it has been previously reported in cancer tissue (COSMIC database id COSM1643037). Hence, we chose to test this variant call with amplicon sequencing. By comparing the variant allele fractions in the original panel data and amplicon sequencing data (S7 Table), we found that the allele fractions were in agreement for the *STAT3* variant (fold change only 0.76–0.85 in amplicon sequencing) but not for the *ITPR3* variant (fold change 0,06–0,075 in amplicon sequencing). Moreover, the *ITPR3* variant was found in amplicon sequencing data of the reference-DNA and in a cell-line control DNA close to the same allele fraction as in the tested CD8+ cell DNAs. These results indicate that the *STAT3* variants are true, while the *ITPR3* C>T variant is a likely artifact, and support the use of stringent filtering to avoid false positives.

### Synonymous somatic mutations

We carried out an additional analysis of synonymous somatic variants in order to include possible effects on RNA structure and other effects not examined by our primary analysis of nonsynonymous mutations. It should be noted that we targeted the coding region of each gene, untranslated regions were not included. After applying the same filters to synonymous variants as we used for the nonsynonymous variants, a total of 72 synonymous somatic mutations were detected in genes expressed in CD8+ cells. Out of these, 43 were found in cases and 29 in controls with no statistical difference in number between the groups (Mann-Whitney U-test p = 0.25). A full list of the detected synonymous mutations is shown in S8 Table.

A clustering of somatic synonymous mutations was detected in the *MAP3K12* gene (Poisson test p = 0.00011), with a total of 4 synonymous mutations, 2 in cases and 2 in controls. All four had the same recurring mutation, NM_001193511:c.279A>G, which was novel. The general purpose CADD algorithm did not predict this mutation to be deleterious (CADD PHRED score 9.024), nor did the algorithm TraP, specialized in synonymous variants, classify it as damaging (TraP score 0.038). However, we also utilized the SURF RNA structural predictivity index that combines different secondary structure effects into one summary metric, and this algorithm did predict the mutation to be deleterious. The somatic *MAP3K12* synonymous mutation had a SURF PHRED score of 21.46, which is inside the 99^th^ percentile and is higher than the scores obtained by the SURF authors for nine known pathogenic mutations affecting RNA structure [36]. This suggests that the synonymous mutation may have a deleterious effect on the stability of MAP3K12 RNA, which is a gene associated with JNK signaling [37]. Although a few singleton mutations had high SURF scores S8 Table), no other genes showed significant (p<0.01) clustering of synonymous mutations.

## Discussion

We had previously shown that it is possible to detect somatic mutations in about 60% of MS patient blood samples, and that the mutations were mostly (85%) discovered in CD8+ cells in comparison to other, CD4+ or CD19+ cells. In the present work, we thus chose to concentrate on the CD8+ cell fraction and included age- and sex-matched controls to assess whether the finding is MS specific, or generalizes to the population at large. Moreover, we were able to perform especially deep sequencing, reaching coverages greater than 2000x, which enabled a sensitive screen of low allelic fraction events. We found 225 nonsynonymous somatic mutations in genes expressed in CD8+ cells and these were distributed evenly between MS patients and healthy controls, with each sample carrying at least one (median 4 mutations in both groups). Including also synonymous and non CD8+ expressed gene variants in the target area there were a total of 652 high-confidence somatic mutations (median and mean numbers of mutations per subject were 13.5 and 15.5).

In order to understand what would be the expected (theoretical) number of detectable mutations in our experiment, we need to consider some technical issues regarding the number of cells and detectable clones. We used 1000 ng of DNA, which corresponds to 167,000 individual cells (6 pg of DNA per cell), of which ≈90% (150,000) were CD8+ cells after the immunomagnetic purification scheme. Our median sequencing depth 2349x, due to the excess of cells, produces essentially haploid sequence from ca. 2100 CD8+ cells (90% of total cells). Usually about half of the blood CD8+ cells are naïve cells, with small clone sizes [38], not detectable with our methodology. The other half (1050 cells) constitutes memory CD8+ cells, which have larger, detectable clones. The mean number of reads in the detected variants was 19.3. Using the mean number of reads as a surrogate for the mean clone size, the estimated mean number of CD8+ memory clones would be 54 (1050/19.3) with large variation in size. Given the non-Gaussian distribution of T cell clones that fits with power law [38, 39] the estimated 54 clones is likely the maximum number of detectable clones in our experiment.

Somatic mutations start to occur and accumulate since the first cell division of the fertilized egg. A recent analysis utilizing individual DNA molecule sequencing of hematopoietic stem cell/progenitor cell colonies and circulating granulocytes from different ages suggest that mutational burden increases linearly with age [40] Based on these data (Figs 2B and 3P in [40]) the expected number of substitutions and small indels in the diploid genome (6200 Mb) at the age of 35 would be ≈880 (35 yrs was the mean age of our donors). The expected number of mutations per cell in our haploid target region (5 Mb) would be 0.71 (880 x 5 Mb/6200 Mb). Assuming that the estimated maximum of 54 clones in our experiment would represent extremely independent phylogenies we would arrive to the estimate of 38 detectable mutations per subject (0.71 x 54). The observed numbers in our study, median 13.5 and mean 15.5 mutations per subject are not far from this figure. This estimation gives an idea of the magnitude of the expected number of mutations. There are several factors that make accurate estimation difficult, but these do not necessarily change the order of magnitude of the expected number of mutations. Shared phylogenies of the clones is very likely, which would result in lower number of independent clones and mutations. The mutation rate of CD8+ cells is currently not known and it may be higher than the rates observed so far in hematopoietic stem cells and granulocytes [40]. Additionally, it should be noted that our variant calling scheme was conservative with a bias towards false negatives rather than false positives.

Most of the discovered mutations were predictably deleterious (Table 2) and many of the mutations confer proliferation and/or survival advantage. Of notable interest were the somatic mutations detected in the *STAT3* gene, most of which (4 out of 6 mutations) have been shown to cause an activating phenotype which affects growth and apoptosis properties of T lymphocytes [10, 41]. In our results, *STAT3* was the gene containing more nonsynonymous somatic mutations than any other gene, showing a statistically significant enrichment. It is likely that the clones that carry *STAT3* mutations have a growth advantage over other clones, increasing their size in the sequenced sample, and thus bring the somatic mutation closer to detection threshold more often than mutations in many other genes. It is of note that the STAT3*D661Y mutation found in one MS patient was also previously discovered in our pilot study in another patient [21]. We are collecting a larger dataset for analyzing the frequencies of somatic *STAT3* variants in MS patients and controls.

The other genes that stood out in the number of enriched nonsynonymous mutations were *SMARCA2*, *SOCS1*, *DNMT3A* and *PPP3CA*. The *SMARCA2* (also known as *BRAHMA*, *BRM*) gene is involved in chromatin remodeling processes and is considered a tumor suppressor gene that is very often downregulated in various cancers [42]. On the other hand, in pancreatic cancer cells SMARCA2 activates epigenetically STAT3 signaling and may promote metastasis [43]. Somatic mutations in *SMARCA2* have been reported in chronic lymphocytic leukemia [44] and, most typically, in adenoid cystic carcinoma [45]. *SOCS1* is a gene with known regulatory functions in autoimmunity and a tumor suppressor in cancer [46]. STAT3 activation is inhibited by SOCS1 via its binding to receptor-associated tyrosine kinases JAK2 and TYK2 [47]. *DNMT3A* codes a methyltransferase protein that is essential for establishing normal epigenetic DNA methylation patterns. Somatic mutations in *DNMT3A* have been implicated in age-related clonal hematopoiesis, wherein a hematopoietic stem cell has acquired a growth advantage mutation resulting in an increased clonal size of its peripheral blood cell progeny [48]. On the other hand, *DNMT3A* loss-of-function somatic mutations are also known for phenotypic effects more specifically tied to T cell behavior. In CD8+ T cells specifically, defects in *DNMT3A* will cause an accelerated shift towards a long-living memory phenotype [49]. *PPP3CA* encodes calcineurin-A, which mediates one of the three major signaling pathways of T cell receptor signaling [50]. Ca2+–calcineurin signaling activates T-cells by mediating nuclear translocation of nuclear factor of activated T cells (NFAT) and plays a role in the maintenance of tumor cells in T-cell leukemia [51].

One potentially functional and recurring synonymous somatic mutation was found in *MAP3K12* encoding mitogen-activated protein 3-kinase 12, also known as dual leucine zipper kinase (DLK). It acts upstream of c-Jun N-terminal kinase (JNK) and p38 MAP kinases in the JNK signalling pathway. There are multiple functional outcomes of this pathway in different cell types, one of which is the regulation of STAT3 [52]. Amplification of the *MAP3K12* locus has been reported in non-Hodgkin’s lymphomas [53].

The most commonly mutated genes we found in blood CD8+ cells differ as compared to the top5 mutated genes reported in sun-exposed normal skin (NOTCH1, NOTCH2, FAT1, TP53, NOTCH3) and normal oesophagus (NOTCH1, TP53, NOTCH2, FAT1, NOTCH3) [54, 55]. This is consistent with the view that the cell type and its’ environment shape the mutations that provide selection advantage. It is of note that the same mutations found in non-neoplastic cells also occur commonly in cancers of the same cell types. The mutations common in normal skin and oesephagus are common in respective cancers [54, 55] and the commonest mutated genes we found in CD8+ cells have all been implicated in hematological malignancies.

It is interesting to consider whether some of the detected somatic mutations might have a role in disease. They can theoretically add some level of cancer risk, as 1/5 of the mutations have been found in cancer cells, even if they likely drive only a limited growth potential in most people, when normal T-cell apoptosis occurs. The mutations were detected in CD8+ cells, and because CD8+ T cells are a potent effector of the immune system, a link to autoimmune disorders is also possible. In our data the somatic mutations were equally distributed between MS patients and healthy controls. However, the antigen specificities and general phenotypic features of the mutated CD8+ clones may be differ in MS patients as compared to controls. An autoreactive clone with an activating somatic mutation would offer a potential mechanistic explanation for a chronic disease like MS. Previously, using the HPRT reporter assay it was shown that MS-patients’ mutated T-lymphocyte clones proliferated in response to myelin antigen [18]. Future work is needed to address this hypothesis.

The sequencing depths of over 2000x that we attained in this research call for special care in handling potential false positive errors that may arise in the sample preparation and sequencing processes. Misclassification of low frequency technical artefacts as reportable variants becomes more likely at low allelic fractions. The sequencing artefacts can arise from several different biochemical processes affecting DNA while it is being processed in the laboratory, such as heating induced spontaneous deamination, base oxidization caused by energy from ultrasonic shearing, or damage from free oxygen radicals [56]. To combat these, we applied several strict filters in our variant calling procedure, considering different false positive classes, and aimed to emphasize specificity over sensitivity. One of the more impactful filters employed in this work was based on calculating the data-derived empirical noise frequency at different possible trinucleotide contexts. As diverse biochemical processes can cause different types of error signatures, this allowed us to require higher levels of evidence at locations where a false positive risk was more present, without a priori knowledge of the error generating processes our library preparation procedure would most induce. We found C>T/G>A substitutions to be highly noisy at low allelic fractions in our sequencing data, especially when encountered in a CpG context. Our pipeline automatically corrected for this by being extra strict at such loci, at the possible cost of missing some true positives. Thermocycling (such as in PCR) induced C>T/G>A errors have been reported previously in the literature, in fact forming 97% of errors in one study [31], which is in line with our data. Furthermore, we used amplicon sequencing to confirm that a repeating false positive of the C>T/G>A was correctly classified, and that the *STAT3* somatic mutations in our MS patients were actual true positives. Other experiments may witness a different base substitution error profile depending on the specific library preparation conditions they employ [56].

As we sequenced a panel of genes chosen because of their association to immunity and cancer, we were not able to run generic enrichment analyses of biological functions using our result variant list, and this is a limitation of the study—the somatic mutations we detected are found in immunity and cancer related genes by design. However, inside the immunity context, STAT3-signaling emerged as one clear pathway possibly affected. Moreover, the repeated hits in genes known to give CD8+ T cells a growth and survival advantage suggest that these mutations may have functional consequences as algorithms predict. As there was an association in our data between the number of mutations detected and the sequencing depth reached by a sample, it is reasonable to expect that even deeper sequencing would increase the number of discovered deleterious somatic mutations and potentially dysregulated clones, even in normal healthy adults such as the control subjects in this study.

In conclusion, our results demonstrate that somatic mutations are present both in MS patients’ and controls blood CD8+ T cells and that they cluster on certain genes such as STAT3. Somatic mutations can be important modifiers of the characteristics of CD8+ T cell clones and may result in dysregulated immune responses. The role of somatic mutations in driving autoimmune responses is presently unclear but given the abundance of mutated clones deserves further attention.

## Supporting information

S1 Fig Sequencing depths. The per sample depths obtained in sequencing.
(PDF)

S2 Fig Age effect. Age vs number of exonic somatic mutations for all samples.
(PDF)

S1 Table Patient details. Patients’ demographic features.
(XLSX)

S2 Table Control details. Control participants’ demographic features.
(XLSX)

S3 Table Panel gene list. List of genes included in the NGS capture panel.
(DOCX)

S4 Table Sequence context noise. Mean background noise tabulated by sequence context and base change.
(XLSX)

S5 Table Variant calling filters. List of filtering criteria used during somatic variant calling.
(XLSX)

S6 Table Nonsynonymous mutations. All nonsynonymous somatic mutations detected in the study.
(XLSX)

S7 Table Amplicon counts. Targeted amplicon sequencing results.
(XLSX)

S8 Table Synonymous mutations. The synonymous mutations detected in the study.
(XLSX)
