# Supplementary figures and images for: CD8+ cell somatic mutations in multiple sclerosis patients and controls—Enrichment of mutations in STAT3 and other genes implicated in hematological malignancies
Source: PLoS One. 2021 Dec 7;16(12):e0261002. doi: 10.1371/journal.pone.0261002 (PMC8651110; doi:10.1371/journal.pone.0261002)

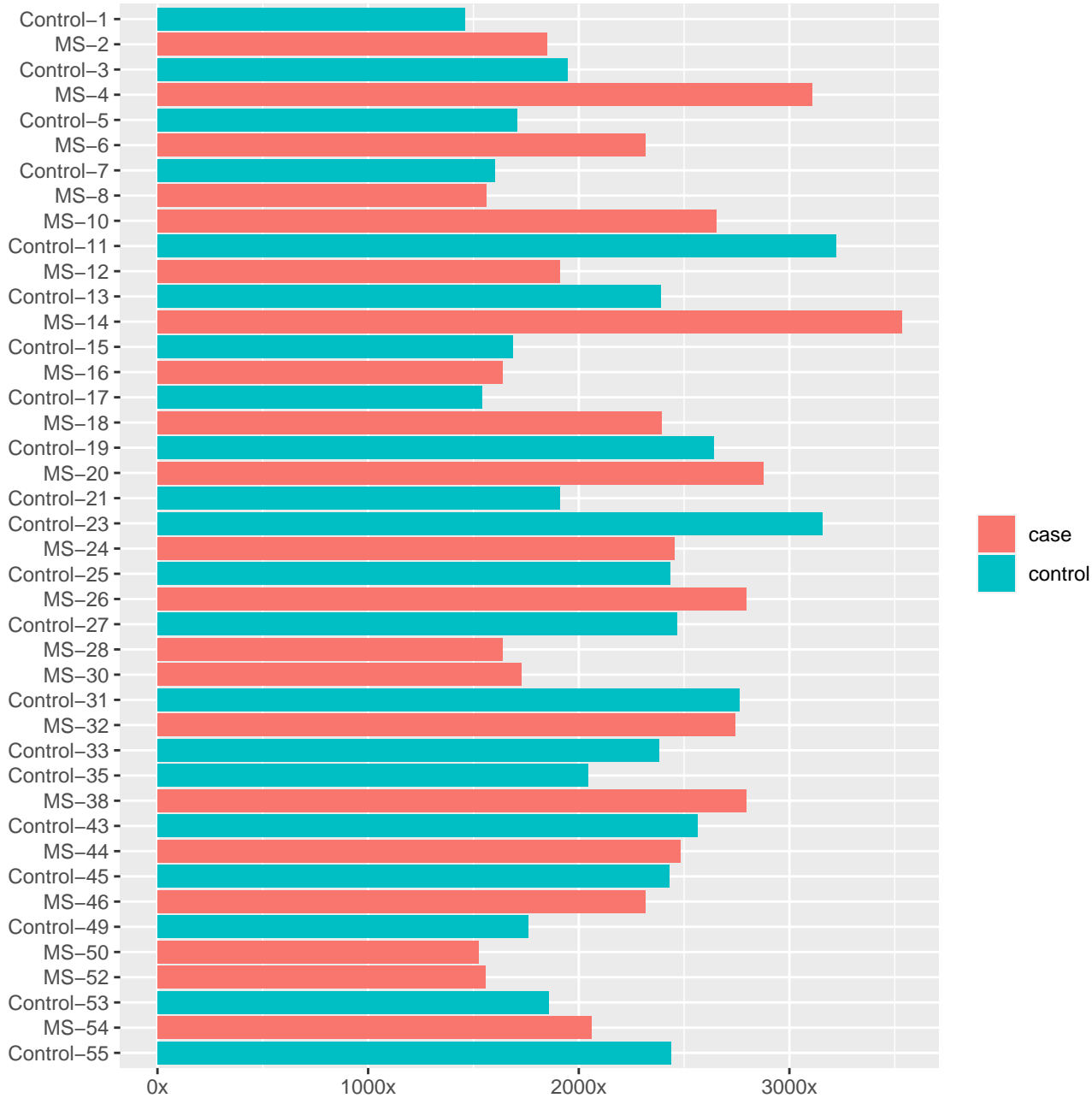

Supplement: S1 Fig — The per sample depths obtained in sequencing. (PDF) [file pone.0261002.s001.pdf]

Age vs Number of Exonic Somatic Mutations per Sequencing Depth

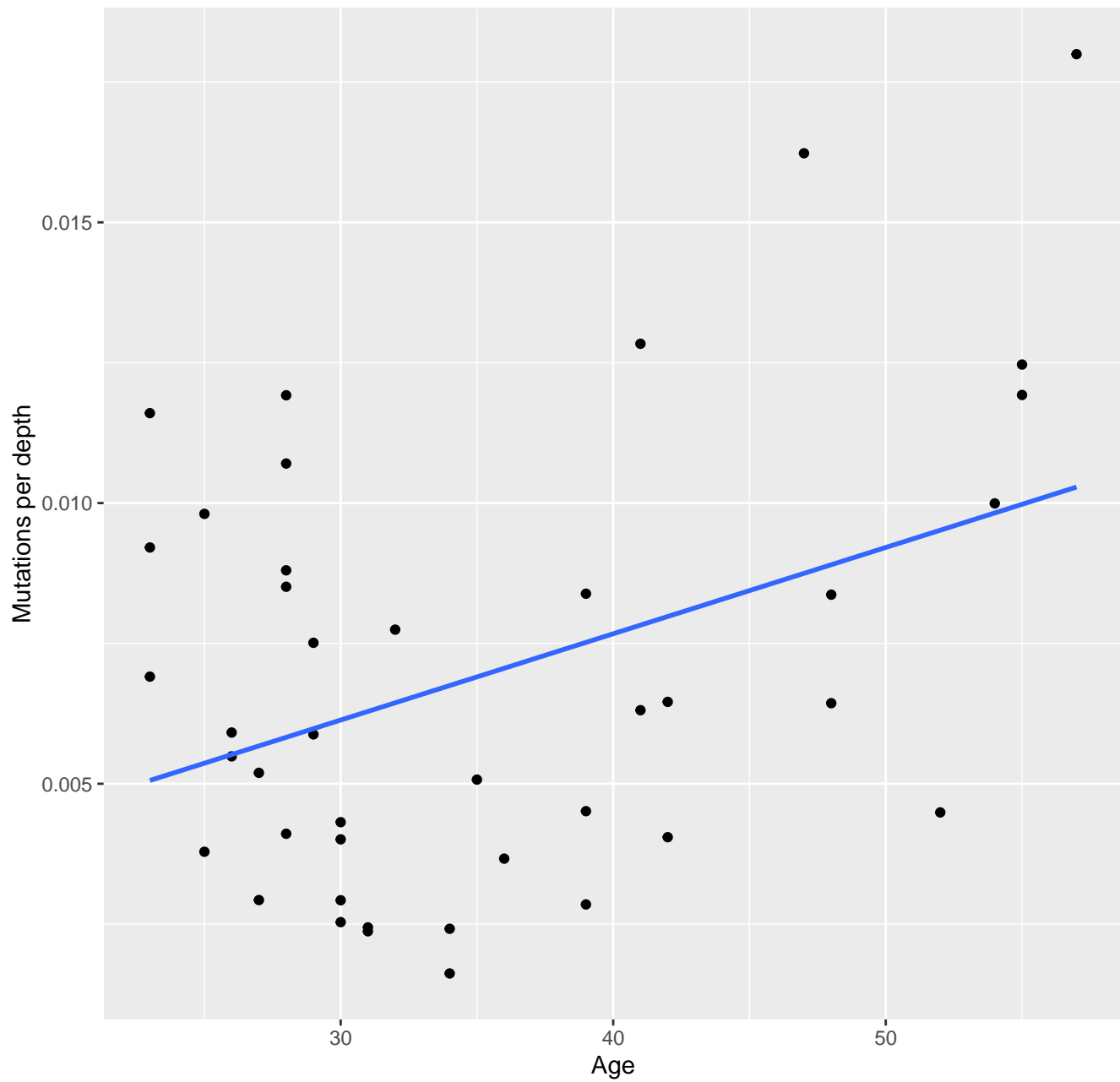

Supplement: S2 Fig — Age vs number of exonic somatic mutations for all samples. (PDF) [file pone.0261002.s002.pdf]
