# Supplementary material for: CD8+ cell somatic mutations in multiple sclerosis patients and controls—Enrichment of mutations in STAT3 and other genes implicated in hematological malignancies
Source: PLoS One. 2021 Dec 7;16(12):e0261002. doi: 10.1371/journal.pone.0261002 (PMC8651110; doi:10.1371/journal.pone.0261002)
Supplement: S3 Table — List of genes included in the NGS capture panel. (DOCX) [file pone.0261002.s005.docx]

**Supplementary Table 3. Immunopanel-2524 gene list.**

A2M

A2ML1

AAMP

ABCA1

ABCA13

ABCB1

ABCB7

ABCF1

ABCG1

ABCG5

ABCG8

ABL1

ACAP1

ACD

ACE

ACE2

ACHE

ACOXL

ACP5

ACTB

ACTN1

ACVRL1

ADA

ADAM10

ADAM17

ADAM8

ADAMTS14

ADAR

ADCY1

ADCY2

ADCY3

ADCY4

ADCY5

ADCY6

ADCY7

ADCY8

ADCY9

ADIPOQ

ADRB2

ADRBK1

ADRBK2

AEBP1

AEBP2

AFF3

AGBL2

AGER

AGFG1

AHI1

AHR

AHSG

AICDA

AIM2

AIMP1

AIRE

AK1

AK2

AKAP10

AKNA

AKT1

AKT1S1

AKT2

AKT3

ALAS2

ALCAM

ALDOA

ALK

AMFR

ANGPT1

ANK1

ANKRD17

ANKRD26

ANKRD30A

ANKRD55

ANO6

ANP32B

ANPEP

ANXA1

ANXA2

ANXA3

ANXA4

ANXA6

AOC3

AP1S3

AP3B1

AP3D1

AP4E1

APCS

APOA1

APOBEC3A

APOBEC3B

APOBEC3G

APOH

APOL1

AQP3

ARAF

ARAP1

ARF6

ARG1

ARHGAP15

ARHGAP8/PRR5-ARHGAP8

ARID3A

ARID5B

ARL5B

ARRB1

ARRB2

ASCC3

ASXL1

ATF2

ATF3

ATF4

ATG12

ATG16L1

ATG5

ATG7

ATG9A

ATM

ATP11A-AS1

ATP6V0D2

ATP7B

ATRIP

ATRX

AVP

AXL

AZI2

B2M

B3GNT2

BACH2

BANK1

BATF

BAX

BCAM

BCAP31

BCAR1

BCL10

BCL11B

BCL2

BCL2A1

BCL2L1

BCL2L11

BCL3

BCL6

BCOR

BCORL1

BDKRB1

BDKRB2

BDNF

BECN1

BEND5

BEX2

BEX4

BEX5

BGN

BID

BIRC2

BIRC3

BIRC5

BLK

BLM

BLNK

BLOC1S6

BMP2

BMX

BOP1

BPI

BRAF

BRCC3

BSG

BST1

BST2

BTBD11

BTG1

BTG2

BTK

BTLA

BTN3A1

BTN3A2

BTN3A3

C10orf55

C15orf41

C1orf106

C1QA

C1QB

C1QBP

C1QC

C1QL1

C1QL2

C1QL3

C1QL4

C1QTNF2

C1QTNF3

C1QTNF4

C1QTNF5

C1QTNF6

C1QTNF7

C1R

C1RL

C1S

C2

C3

C3AR1

C4A

C4B

C4BPA

C4BPB

C5

C5AR1

C5orf30

C6

C7

C8A

C8B

C8G

C9

C9orf47

CAB39

CAB39L

CACNA1A

CACNA1B

CACNA1C

CACNA1D

CACNA1E

CACNA1F

CACNA1G

CACNA1H

CACNA1I

CACNA1S

CACNA2D1

CACNA2D2

CACNA2D3

CACNA2D4

CACNB1

CACNB2

CACNB3

CACNB4

CACNG1

CACNG2

CACNG3

CACNG4

CACNG5

CACNG6

CACNG7

CACNG8

CALCA

CALCOCO2

CALM1

CAMK1

CAMK2A

CAMK2B

CAMK2D

CAMK2G

CAMK4

CAMKK2

CAMLG

CAMP

CANX

CAPN1

CAPN2

CAPRIN1

CARD11

CARD14

CARD16

CARD18

CARD6

CARD8

CARD9

CASKIN1

CASP1

CASP10

CASP12

CASP2

CASP3

CASP4

CASP5

CASP6

CASP7

CASP8

CASP9

CAV1

CBL

CBLB

CBLC

CCBE1

CCDC88A

CCL1

CCL11

CCL13

CCL14

CCL15

CCL15-CCL14

CCL16

CCL17

CCL18

CCL19

CCL2

CCL20

CCL21

CCL22

CCL23

CCL24

CCL25

CCL26

CCL27

CCL28

CCL3

CCL3L1

CCL3L3

CCL4

CCL4L2

CCL5

CCL7

CCL8

CCNA2

CCND1

CCND2

CCND3

CCNE1

CCNT1

CCR1

CCR10

CCR2

CCR3

CCR4

CCR5

CCR6

CCR7

CCR8

CCR9

CCRL2

CCRN4L

CD101

CD109

CD14

CD151

CD160

CD163

CD164

CD164L2

CD180

CD19

CD1A

CD1B

CD1C

CD1D

CD1E

CD2

CD200

CD200R1

CD200R1L

CD207

CD209

CD22

CD226

CD244

CD247

CD248

CD27

CD274

CD276

CD28

CD2AP

CD2BP2

CD300A

CD300C

CD300E

CD300LB

CD300LF

CD300LG

CD302

CD320

CD33

CD34

CD36

CD37

CD38

CD3D

CD3E

CD3EAP

CD3G

CD4

CD40

CD40LG

CD44

CD46

CD47

CD48

CD5

CD52

CD53

CD55

CD58

CD59

CD5L

CD6

CD63

CD68

CD69

CD7

CD70

CD72

CD74

CD79A

CD79B

CD80

CD81

CD82

CD83

CD84

CD86

CD8A

CD8B

CD9

CD93

CD96

CD99

CD99L2

CDAN1

CDC25B

CDC42

CDCA7

CDH23

CDH3

CDH5

CDK2

CDK4

CDK6

CDK9

CDKN1A

CDKN2A

CDKN2B

CEACAM1

CEACAM3

CEACAM5

CEACAM6

CEACAM8

CEBPA

CEBPB

CEBPD

CEBPE

CECR1

CENPO

CEP57

CFB

CFD

CFH

CFHR1

CFHR2

CFHR3

CFHR4

CFHR5

CFI

CFLAR

CFP

CFTR

CHAT

CHD2

CHD7

CHEK1

CHGA

CHL1

CHUK

CIITA

CISH

CKLF

CLCF1

CLEC10A

CLEC12A

CLEC16A

CLEC1B

CLEC4A

CLEC4C

CLEC4D

CLEC4E

CLEC4M

CLEC5A

CLEC6A

CLEC7A

CLEC9A

CLECL1

CLIP1

CLIP2

CLNK

CLPB

CLTC

CLU

CMA1

CMKLR1

CMTM1

CMTM2

CMTM3

CMTM4

CMTM5

CMTM6

CMTM7

CMTM8

CNKSR2

CNN3

CNOT3

CNOT4

CNOT8

CNPY3

CNRIP1

CNTF

CNTFR

CNTLN

COCH

COG6

COL6A1

COLEC11

COLEC12

COPA

COPS5

COPS8

CORO1A

CORO2A

COX4I2

COX5B

CPB2

CR1

CR1L

CR2

CRADD

CREB1

CREBBP

CRIP2

CRK

CRKL

CRLF1

CRLF2

CRLF3

CRP

CRYAB

CSF1

CSF1R

CSF2

CSF2RA

CSF2RB

CSF3

CSF3R

CSH1

CSH2

CSK

CSMD1

CSN2

CST7

CTC1

CTCF

CTF1

CTLA4

CTNNAL1

CTNNB1

CTNND1

CTPS1

CTSB

CTSC

CTSD

CTSF

CTSG

CTSH

CTSK

CTSL

CTSO

CTSS

CTSV

CTSW

CTSZ

CUL7

CUX1

CX3CL1

CX3CR1

CXCL1

CXCL10

CXCL11

CXCL12

CXCL13

CXCL14

CXCL16

CXCL2

CXCL3

CXCL5

CXCL6

CXCL9

CXCR1

CXCR2

CXCR3

CXCR4

CXCR5

CXCR6

CYBA

CYBB

CYCS

CYLD

CYP24A1

CYSLTR1

CYTIP

CYTL1

DAB2IP

DAG1

DAGLB

DAPK1-IT1

DAXX

DCD

DCHS1

DCLRE1C

DCN

DDAH1

DDI1

DDIT3

DDIT4

DDR1

DDX1

DDX21

DDX3X

DDX41

DDX58

DEFA1

DEFA3

DEFA4

DEFA5

DEFA6

DEFB1

DEFB103A

DEFB103B

DEFB105A

DEFB106A

DEFB119

DEFB123

DEFB4A

DHCR24

DHX36

DHX58

DHX9

DICER1

DIDO1

DIS3

DKC1

DKKL1

DLG1

DLK1

DMBT1

DNM1L

DNMT3A

DNMT3B

DOCK2

DOCK4

DOCK8

DOK3

DPP4

DPY19L2

DRD2

DTNBP1

DUOX1

DUOX2

DUSP1

DUSP10

DUSP16

DUSP2

DUSP3

DUSP4

DUSP5

DUSP6

DUSP7

DUSP8

DUSP9

E2F1

EBF1

EBF2

EBI3

ECSIT

EDIL3

EDN1

EED

EGF

EGFR

EGLN1

EGLN2

EGLN3

EGR1

EHMT2

EIF2AK2

EIF4B

EIF4E

EIF4E1B

EIF4E2

EIF4EBP1

EIF4EBP2

ELANE

ELAVL1

ELF1

ELF2

ELF4

ELK1

ELK4

ELMO1

ELMOD2

ELP2

ENC1

ENG

ENTPD1

EOMES

EP300

EPAS1

EPB42

EPO

EPOR

EPS15L1

EPS8

EPX

ERAP1

ERBB2IP

ERCC6L2

ERGIC2

ERLIN1

ERN1

ETNK1

ETS1

ETS2

ETV6

ETV7

EVI5

EZH1

EZH2

EZR

F10

F11

F12

F13A1

F13B

F2

F2R

F2RL1

F2RL2

F2RL3

F3

F5

F7

F8

F9

FADD

FADS1

FADS2

FADS3

FAM172BP

FAM46C

FAM53B

FAM84B

FANCA

FANCB

FANCC

FANCD2

FANCE

FANCF

FANCI

FANCL

FANCM

FAS

FASLG

FBXO11

FBXW5

FBXW7

FCAMR

FCAR

FCER1A

FCER1G

FCER2

FCGR1A

FCGR2A

FCGR2B

FCGR2C

FCGR3A

FCGR3B

FCGRT

FCN1

FCN2

FCN3

FCRL1

FCRL3

FCRL5

FCRLA

FER

FERMT3

FFAR2

FGA

FGB

FGF1

FGF10

FGF11

FGF12

FGF13

FGF14

FGF16

FGF17

FGF18

FGF19

FGF2

FGF20

FGF21

FGF22

FGF23

FGF3

FGF4

FGF5

FGF6

FGF7

FGF8

FGF9

FGFBP1

FGFR1

FGFR2

FGFR3

FGFR4

FGG

FGR

FIGF

FLI1

FLNA

FLNB

FLNC

FLT3

FLT4

FOS

FOXA2

FOXK2

FOXN1

FOXO1

FOXO3

FOXP1

FOXP3

FPR1

FPR2

FPR3

FREM1

FRK

FSCN1

FSTL1

FUT3

FXR1

FYB

FYN

FZD1

FZD4

G3BP1

G6PC

G6PC3

G6PD

GAB1

GAB2

GABARAP

GADD45A

GADD45B

GADD45G

GALC

GALNS

GAS2L3

GAS6

GATA1

GATA2

GATA3

GATA4

GATA6

GBA

GBP1

GBP2

GBP3

GBP4

GBP5

GBP6

GBP7

GFI1

GFI1B

GH1

GH2

GHR

GJA1

GLI1

GLIS2

GLRX

GLRX5

GNA12

GNA13

GNAI1

GNAI2

GNAI3

GNAS

GNB1

GNB2

GNB2L1

GNB3

GNB4

GNB5

GNG10

GNG11

GNG12

GNG13

GNG2

GNG3

GNG4

GNG5

GNG7

GNG8

GNGT1

GNGT2

GNLY

GNS

GOPC

GP1BA

GP1BB

GP2

GP5

GP9

GPI

GPR153

GPR183

GPR33

GPR52

GPRASP1

GPRC5A

GPSM1

GRB2

GRHL2

GRK1

GRK4

GRK5

GRK6

GRK7

GRN

GSK3A

GSK3B

GSR

GSS

GSTP1

GUSB

GYPA

GYPB

GYPC

GYPE

GZMA

GZMB

GZMK

GZMM

HAMP

HAVCR2

HAX1

HBA1

HBA2

HBB

HCK

HCST

HDAC1

HDAC11

HDAC2

HELLS

HERC5

HFE

HFE2

HGF

HGSNAT

HHEX

HIF1A

HIF1AN

HIF3A

HIST1H1B

HIST1H1C

HIST1H1D

HIST1H1E

HIST1H3B

HK1

HMGB1

HMGB2

HMGB3

HMGN2

HMMR

HMOX1

HNRNPL

HOOK1

HOXA11

HOXA9

HOXC5

HP

HPS3

HRAS

HRG

HRH2

HRH4

HSF1

HSP90AA1

HSP90AB1

HSP90B1

HSPA14

HSPA1A

HSPA1B

HSPA1L

HSPA2

HSPA4

HSPA6

HSPA8

HSPB1

HSPBP1

HSPD1

HTN3

HYAL1

HYOU1

ICAM1

ICAM2

ICAM3

ICAM4

ICOS

ICOSLG

ID3

IDH1

IDH2

IDO1

IDS

IDUA

IFI16

IFI27

IFI30

IFI35

IFI44

IFI44L

IFI6

IFIH1

IFIT1

IFIT1B

IFIT2

IFIT3

IFITM1

IFITM2

IFITM3

IFITM5

IFNA1

IFNA10

IFNA13

IFNA14

IFNA16

IFNA17

IFNA2

IFNA21

IFNA4

IFNA5

IFNA6

IFNA7

IFNA8

IFNAR1

IFNAR2

IFNB1

IFNE

IFNG

IFNGR1

IFNGR2

IFNK

IFNW1

IGF1

IGF1R

IGF2R

IGHA1

IGHA2

IGHE

IGHG1

IGHG2

IGHG3

IGHG4

IGHM

IGKC

IGLL1

IGSF8

IKBKAP

IKBKB

IKBKE

IKBKG

IKZF1

IKZF2

IKZF3

IL10

IL10RA

IL10RB

IL11

IL11RA

IL12A

IL12B

IL12RB1

IL12RB2

IL13

IL13RA1

IL13RA2

IL15

IL15RA

IL16

IL17A

IL17B

IL17C

IL17D

IL17F

IL17RA

IL17RB

IL17RC

IL17RD

IL17RE

IL18

IL18BP

IL18R1

IL18RAP

IL19

IL1A

IL1B

IL1F10

IL1R1

IL1R2

IL1RAP

IL1RAPL1

IL1RAPL2

IL1RL1

IL1RL2

IL1RN

IL2

IL20

IL20RA

IL20RB

IL21

IL21R

IL21R-AS1

IL22

IL22RA1

IL22RA2

IL23A

IL23R

IL24

IL25

IL26

IL27

IL27RA

IL2RA

IL2RB

IL2RG

IL3

IL31

IL31RA

IL32

IL33

IL36A

IL36G

IL36RN

IL37

IL3RA

IL4

IL4I1

IL4R

IL5

IL5RA

IL6

IL6R

IL6ST

IL7

IL7R

IL9

IL9R

ILF2

ILF3

IMPDH2

ING4

INHBA

INO80

INPP5D

INS

INSIG1

INSR

INTS12

IP6K1

IQCB1

IQGAP1

IRAK1

IRAK1BP1

IRAK2

IRAK3

IRAK4

IRF1

IRF2

IRF2BP1

IRF3

IRF4

IRF5

IRF6

IRF7

IRF8

IRF9

IRGM

IRS1

ISG15

ISG20

ISL2

ITCH

ITFG1

ITGA1

ITGA2

ITGA2B

ITGA3

ITGA4

ITGA5

ITGA6

ITGAD

ITGAE

ITGAL

ITGAM

ITGAV

ITGAX

ITGB1

ITGB2

ITGB3

ITGB4

ITK

ITPR1

ITPR3

JAGN1

JAK1

JAK2

JAK3

JAM3

JARID2

JAZF1

JMJD7-PLA2G4B

JUN

JUND

KAT2B

KAZALD1

KCNJ8

KCNMA1

KDM4A

KDM6A

KDR

KEAP1

KEL

KHSRP

KIAA0226

KIF21B

KIF23

KIR2DL1

KIR2DL3

KIR2DL4

KIR2DP1

KIR2DS4

KIR3DL1

KIR3DL2

KIR3DX1

KIT

KITLG

KLF1

KLF4

KLHL6

KLK1

KLKB1

KLRB1

KLRC1

KLRC2

KLRC3

KLRD1

KLRK1

KMT2D

KNG1

KPNA1

KRAS

KREMEN2

KRT16

KY

L1CAM

LACC1

LAG3

LAIR1

LAIR2

LAMB4

LAMP1

LAMP2

LAMP3

LAMTOR2

LAMTOR3

LAP3

LAT

LAX1

LBH

LBP

LCAT

LCK

LCN2

LCP1

LCP2

LEAP2

LEF1

LEP

LEPR

LGALS1

LGALS2

LGALS3

LGALS3BP

LGALS4

LGALS8

LGALS9

LGMN

LGR4

LIF

LIFR

LIG1

LIG4

LILRA1

LILRA2

LILRA3

LILRA4

LILRA5

LILRA6

LILRB1

LILRB2

LILRB3

LILRB4

LILRB5

LIPA

LITAF

LPCAT2

LPIN2

LPO

LRBA

LRG1

LRP1

LRRC8A

LRRFIP1

LRRFIP2

LRRK2

LSP1

LST1

LTA

LTA4H

LTB

LTB4R

LTB4R2

LTBR

LTF

LUC7L2

LUM

LY6E

LY75

LY86

LY9

LY96

LYG2

LYN

LYNX1

LYST

LYZ

MAD1L1

MAF

MAFB

MAGT1

MAL

MALT1

MANBA

MAP1LC3C

MAP2K1

MAP2K2

MAP2K3

MAP2K4

MAP2K5

MAP2K6

MAP2K7

MAP3K1

MAP3K11

MAP3K12

MAP3K13

MAP3K14

MAP3K2

MAP3K3

MAP3K4

MAP3K5

MAP3K6

MAP3K7

MAP3K8

MAP4K1

MAP4K2

MAP4K3

MAP4K4

MAP9

MAPK1

MAPK10

MAPK11

MAPK12

MAPK13

MAPK14

MAPK3

MAPK6

MAPK7

MAPK8

MAPK8IP1

MAPK8IP2

MAPK8IP3

MAPK9

MAPKAPK2

MAPKAPK3

MAPKAPK5

MAPT

MARCH5

MARCO

MASP1

MASP2

MAVS

MAX

MB21D1

MBL2

MBP

MBTPS1

MBTPS2

MCAM

MCL1

MCM10

MCM4

MDC1

MDM2

MECOM

MED1

MED12

MEF2B

MEF2C

MEFV

MERTK

MFF

MFGE8

MFI2

MFN1

MFN2

MICA

MICB

MID1

MID2

MIF

MINOS1-NBL1/NBL1

MIR107

MIR10A

MIR10B

MIR1204

MIR1208

MIR122

MIR124-1

MIR125B1

MIR125B2

MIR126

MIR1275

MIR132

MIR133A1

MIR135B

MIR141

MIR145

MIR146A

MIR149

MIR15B

MIR16-1

MIR16-2

MIR181A2

MIR187

MIR200C

MIR208B

MIR21

MIR212

MIR223

MIR23A

MIR23B

MIR302B

MIR3148

MIR372

MIR373

MIR497

MIR499A

MIR517A

MIR517C

MIR548AN

MIR548G

MIRLET7B

MIRLET7C

MKL1

MKNK1

MKNK2

MLPH

MLST8

MME

MMEL1

MMP1

MMP10

MMP11

MMP12

MMP13

MMP14

MMP15

MMP16

MMP17

MMP19

MMP2

MMP20

MMP21

MMP23A

MMP23B

MMP24

MMP25

MMP26

MMP27

MMP28

MMP3

MMP7

MMP8

MMP9

MOGS

MOS

MOV10

MPL

MPO

MPZL3

MR1

MRAS

MRC1

MRC2

MRE11A

MRGPRX2

MS4A1

MS4A3

MS4A5

MSH6

MSR1

MST1

MST1R

MTA1

MTHFD1

MTOR

MUC1

MUL1

MVK

MX1

MX2

MXRA5

MYC

MYD88

MYH9

MYLK

MYO18A

MYO5A

MYSM1

NAGLU

NAIP

NAMPT

NBAS

NBEA

NBEAL2

NBN

NCAM1

NCF1

NCF2

NCF4

NCK1

NCK2

NCKAP1L

NCOR2

NCR1

NCR2

NCR3

NDFIP1

NDUFAF4P4

NDUFS3

NEU1

NF1

NFAT5

NFATC1

NFATC2

NFATC3

NFATC4

NFE2L2

NFIL3

NFKB1

NFKB2

NFKBIA

NFKBIB

NFKBIE

NFKBIL1

NFKBIZ

NFRKB

NGF

NHEJ1

NHP2

NKIRAS1

NKIRAS2

NKX2-3

NKX2-5

NKX3-1

NLK

NLRC3

NLRC4

NLRC5

NLRP1

NLRP10

NLRP11

NLRP12

NLRP13

NLRP14

NLRP2

NLRP3

NLRP4

NLRP5

NLRP6

NLRP7

NLRP8

NLRP9

NLRX1

NMI

NOD1

NOD2

NOL3

NOP10

NOP9

NOS2

NOTCH1

NOTCH2

NOX1

NOX4

NOXA1

NPC1

NPC2

NPEPPS

NPM1

NPTN

NR1H3

NR1H4

NR3C1

NR4A1

NR4A3

NR5A2

NRAS

NRIP1

NT5C3A

NT5E

NTF3

NTF4

NTN1

NTRK1

NTRK2

NUDCD3

NUMB

NUMBL

NUP153

NUP214

NXN

OAS1

OAS2

OAS3

OASL

OLFM4

OPTN

OR1D5

ORAI1

OSM

OSMR

OTUB1

OTUB2

OTUD5

OTUD7B

OXNAD1

P2RX7

P2RY10

P2RY14

P2RY8

P4HTM

PACSIN1

PADI4

PAFAH1B1

PAFAH1B2

PAFAH1B3

PAFAH2

PAK1

PAK2

PAPD5

PARD3

PARK2

PARP1

PAX2

PAX5

PCBP1

PCBP2

PDCD1

PDCD1LG2

PDE3B

PDGFA

PDGFB

PDGFRA

PDGFRB

PDK1

PDPK1

PDS5B

PDSS2

PEG3

PELI1

PELI2

PELI3

PF4

PF4V1

PFKL

PFKM

PGK1

PGLYRP1

PGLYRP2

PGLYRP3

PGLYRP4

PGM3

PHF6

PHGDH

PIAS1

PIAS2

PIAS3

PIAS4

PIEZO1

PIGA

PIK3AP1

PIK3C3

PIK3CA

PIK3CB

PIK3CD

PIK3CG

PIK3R1

PIK3R2

PIK3R3

PIK3R5

PILRA

PIM1

PIN1

PITPNM2

PKLR

PKN1

PLA2G4A

PLA2G4B

PLA2G4C

PLA2G4D

PLA2G4E

PLA2G4F

PLA2G7

PLA2R1

PLAA

PLAG1

PLAT

PLAU

PLAUR

PLCB1

PLCB2

PLCB3

PLCB4

PLCG1

PLCG2

PLCL2

PLEC

PLEK

PLEKHG5

PLG

PLK1

PLK3

PLSCR1

PLTP

PLXDC1

PLXNA4

PLXNC1

PMAIP1

PML

PMS2

PNP

POLE

POLR1C

POLR1D

POLR2E

POLR2F

POLR2H

POLR2K

POLR2L

POLR3A

POLR3B

POLR3C

POLR3D

POLR3E

POLR3F

POLR3G

POLR3GL

POLR3H

POLR3K

POMC

POT1

POU2AF1

POU2F2

PPARG

PPARGC1A

PPARGC1B

PPBP

PPIA

PPIL4

PPM1A

PPM1B

PPM1D

PPP1CA

PPP1CC

PPP3CA

PPP3CB

PPP3CC

PPP3R1

PPP3R2

PPP4C

PPP5C

PPP5D1

PRDM1

PRDX5

PRDX6

PREX1

PRF1

PRG2

PRKAA1

PRKAA2

PRKACA

PRKACB

PRKACG

PRKCA

PRKCB

PRKCD

PRKCE

PRKCG

PRKCH

PRKCQ

PRKCZ

PRKDC

PRKRA

PRKX

PRL

PRLR

PRMT1

PRNP

PROC

PROCR

PROM1

PROS1

PRPF40B

PRPF8

PRRC2A

PRSS16

PRSS23

PRTN3

PSAP

PSG1

PSIP1

PSMA7

PSMB10

PSMB5

PSMB6

PSMB7

PSMB8

PSMB9

PSME1

PSME2

PSME3

PSMF1

PSMG1

PSTPIP1

PTAFR

PTCH1

PTEN

PTGER4

PTGES

PTGES2

PTGFRN

PTGS2

PTK2

PTK2B

PTMA

PTPN11

PTPN2

PTPN22

PTPN5

PTPN6

PTPN7

PTPRC

PTPRCAP

PTPRE

PTPRJ

PTPRR

PTPRU

PTX3

PURA

PUS1

PVR

PVRL1

PVRL2

PVT1

PXK

PXN

PXT1

PYCARD

PYDC1

PYHIN1

RAB11A

RAB27A

RAB8A

RAC1

RAC2

RAC3

RAD21

RAD23A

RAD50

RAD51B

RAET1E

RAET1G

RAET1L

RAF1

RAG1

RAG2

RANBP9

RAP1A

RAP1B

RAPGEF2

RARRES2

RASA1

RASA2

RASGEF1B

RASGRF1

RASGRF2

RASGRP1

RASGRP2

RASGRP3

RASGRP4

RB1

RBBP4

RBBP7

RBCK1

RBM11

RBM8A

RBPJ

RCAN1

RCAN3

RCOR1

RECQL4

REG3G

REG4

REL

RELA

RELB

REST

RETNLB

RFTN1

RFX1

RFX5

RFXANK

RFXAP

RGMB

RGS1

RGS14

RGS2

RHAG

RHBDF2

RHCE

RHD

RHEB

RHOA

RHOG

RHOH

RICTOR

RIPK1

RIPK2

RIPK3

RIT1

RLTPR

RMI2

RMRP

RNASE7

RNASEH2A

RNASEH2B

RNASEH2C

RNASEL

RNF125

RNF135

RNF168

RNF31

RNF41

RNF5

ROCK1

ROCK2

ROR1

RORA

RORC

RPA1

RPL10

RPL11

RPL15

RPL19

RPL35A

RPL5

RPS10

RPS15

RPS15AP6

RPS16

RPS17

RPS19

RPS2

RPS24

RPS26

RPS28

RPS29

RPS6

RPS6KA1

RPS6KA2

RPS6KA3

RPS6KA4

RPS6KA5

RPS6KA6

RPS6KB1

RPS6KB2

RPS7

RPSA

RPTOR

RRAGA

RRAGB

RRAGC

RRAGD

RRAS

RRAS2

RSAD2

RTEL1

RTKN2

RUNX1

RUNX3

RUSC1

RXRA

S100A10

S100A12

S100A8

S100A9

S1PR1

SAA1

SAA2

SAMD12

SAMHD1

SAMSN1

SARM1

SATB2

SBDS

SBF2

SBK1

SCAF11

SCAMP5

SCARB1

SCARB2

SCARF1

SCGB3A1

SCML1

SCN5A

SDC1

SDC3

SDC4

SDF2

SDF2L1

SDK2

SDR42E1

SEC23B

SELE

SELL

SELP

SELPLG

SEMA3A

SEMA4D

SEMA7A

SENP2

SERPINA1

SERPINA5

SERPINB2

SERPINB9

SERPINC1

SERPIND1

SERPINE1

SERPINF2

SERPING1

SETBP1

SF3A1

SF3B1

SFRP4

SFRP5

SFTPA1

SFTPA2

SFTPD

SGK1

SGSH

SH2B3

SH2D1A

SH2D1B

SH3BP2

SHANK1

SHARPIN

SHC1

SHC2

SHC3

SHC4

SIAH1

SIAH2

SIGIRR

SIGLEC1

SIGLEC10

SIGLEC11

SIGLEC15

SIGLEC5

SIGLEC6

SIGLEC7

SIGLEC8

SIGLEC9

SIKE1

SIRPA

SIRT1

SIVA1

SKIV2L

SLA

SLAMF1

SLAMF6

SLAMF7

SLAMF8

SLAMF9

SLC11A1

SLC15A4

SLC19A2

SLC1A4

SLC22A3

SLC25A38

SLC29A3

SLC2A4RG

SLC35C1

SLC37A4

SLC3A2

SLC40A1

SLC44A1

SLC44A2

SLC46A1

SLC4A1

SLC6A12

SLC7A11

SLC7A5

SLC9A8

SLX4

SMAD3

SMAD4

SMAD6

SMAD7

SMARCA2

SMARCA4

SMARCAL1

SMARCE1

SMC1A

SMC3

SMPD1

SNAP23

SNCA

SNX27

SOAT2

SOCS1

SOCS2

SOCS3

SOCS4

SOCS5

SOCS6

SOCS7

SOD1

SOD3

SOS1

SOS2

SP110

SP140

SPAG11A

SPAG7

SPHK1

SPI1

SPINK2

SPINK5

SPN

SPON2

SPP1

SPRED1

SPRED2

SPRED3

SPRY1

SPRY2

SPRY4

SPTA1

SPTB

SQSTM1

SRC

SREBF1

SREBF2

SRF

SRP72

SRSF2

SRXN1

ST6GAL1

ST6GALNAC1

STAB1

STAG1

STAG2

STAM

STAM2

STAP2

STAT1

STAT2

STAT3

STAT4

STAT5A

STAT5B

STAT6

STIM1

STK11

STK3

STK4

STMN1

STRADA

STUB1

STX11

STXBP2

SUCNR1

SUGT1

SUOX

SUZ12

SWAP70

SYDE2

SYK

SYNGR1

SYP

TAB1

TAB2

TAB3

TACR1

TAGAP

TAL1

TANK

TAOK1

TAOK2

TAOK3

TAP1

TAP2

TAPBP

TAX1BP1

TAZ

TBK1

TBKBP1

TBL1XR1

TBX1

TBX21

TCEB1

TCEB2

TCF3

TCF4

TCF7

TCIRG1

TCL1A

TCL1B

TCN2

TDP2

TEC

TECPR1

TEK

TENM3

TERC

TERF1

TERF2

TERT

TET1

TET2

TEX41

TFAP2A

TFPI

TFR2

TFRC

TGFB1

TGFB2

TGFB3

TGFBI

TGFBR1

TGFBR2

TGFBR3

TGM2

THBD

THBS1

THPO

THRB

THY1

TIAM1

TICAM1

TICAM2

TIMMDC1

TIMP1

TINF2

TIRAP

TKTL2

TLN1

TLR1

TLR10

TLR2

TLR3

TLR4

TLR5

TLR6

TLR7

TLR8

TLR9

TMC6

TMC8

TMED7

TMEM126A

TMEM173

TMEM220

TMEM30A

TMIGD2

TNF

TNFAIP3

TNFAIP8L2

TNFRSF10A

TNFRSF10B

TNFRSF10C

TNFRSF10D

TNFRSF11A

TNFRSF12A

TNFRSF13B

TNFRSF13C

TNFRSF14

TNFRSF17

TNFRSF18

TNFRSF1A

TNFRSF1B

TNFRSF25

TNFRSF4

TNFRSF8

TNFRSF9

TNFSF10

TNFSF11

TNFSF12

TNFSF13

TNFSF13B

TNFSF14

TNFSF15

TNFSF4

TNFSF8

TNFSF9

TNIP1

TNIP3

TNK1

TOLLIP

TOM1

TOMM70A

TONSL

TP53

TP63

TP73

TPD52

TPH1

TPI1

TPO

TPP2

TPSB2

TPST1

TRABD2A

TRAC

TRADD

TRAF1

TRAF2

TRAF3

TRAF3IP1

TRAF3IP2

TRAF4

TRAF5

TRAF6

TRAF7

TRAFD1

TRAIP

TRAT1

TRAV30

TRDJ1

TREH

TREM1

TREM2

TREML2

TREX1

TRIB2

TRIB3

TRIM13

TRIM14

TRIM15

TRIM21

TRIM22

TRIM23

TRIM24

TRIM25

TRIM26

TRIM27

TRIM28

TRIM32

TRIM36

TRIM37

TRIM38

TRIM42

TRIM45

TRIM47

TRIM49

TRIM5

TRIM50

TRIM55

TRIM56

TRIM58

TRIM6

TRIM60

TRIM61

TRIM62

TRIM63

TRIM65

TRIM66

TRIM67

TRIM7

TRIM71

TRIM8

TRIM9

TRIP6

TRNT1

TRPM2

TRPV2

TSC1

TSC2

TSC22D3

TSFM

TSLP

TSPYL2

TSR2

TTC37

TTC7A

TUBB1

TUFM

TXNDC11

TXNIP

TYK2

TYRO3

TYROBP

TYW1

U2AF1

U2AF2

UBASH3A

UBC

UBD

UBE2N

UBQLN1

UBR5

UCHL1

UCP2

ULBP1

ULBP2

ULBP3

ULK1

ULK2

ULK3

UMODL1

UNC119

UNC13D

UNC5CL

UNC93B1

UNG

USB1

USP18

USP2

USP4

USP44

USP7

WAS

WASF1

WASF3

WASL

VASP

VAV1

VAV2

VAV3

VCAM1

VCAN

WDFY1

WDFY3-AS2

WDFY4

VDR

WDR34

WDR62

VEGFA

VEGFB

VEGFC

VENTX

WFDC12

VHL

WHSC1

WIPF1

VLDLR

VMP1

WNT2B

WNT3A

WNT9B

VPREB1

VPREB3

VPS13B

VPS33B

VPS45

WRAP53

VSIG1

VSTM2B

WT1

VTRNA2-1

VWF

WWOX

XBP1

XCL1

XCL2

XCR1

XDH

XIAP

XPO1

XRCC2

XRCC5

XRCC6

YARS2

YBX1

YJEFN3

YWHAE

YWHAZ

YY1

ZAP70

ZBP1

ZBTB16

ZBTB24

ZC2HC1A

ZFP36L1

ZMIZ1
